# 3-[2-(1,3-Benzothia­zol-2-ylsulfan­yl)eth­yl]-1,3-oxazolidin-2-one

**DOI:** 10.1107/S1600536810034264

**Published:** 2010-09-04

**Authors:** Cong-Hui Ma, Xiao-Feng Li, Yan An, Yong-Hong Wen

**Affiliations:** a College of Chemistry and Molecular Engineering, Qingdao University of Science and Technology, Qingdao 266042, People’s Republic of China; b Institute of Marine Materials Science and Engineering, Shanghai Maritime University, Shanghai 201305, People’s Republic of China

## Abstract

The title compound, C_12_H_12_N_2_S_2_O_2_, consists of a benzothia­zole group and a oxazolidin-1-one linked *via* a flexible ethane-1,2-diyl spacer.  The benzothiazole group and the oxazolidine ring are each almost planar [with maximum deviations of 0.007 (2) and 0.044 (3) Å, respectively] and make a dihedral angle of 9.35 (10)°. In the crystal structure, adjacent mol­ecules were connected through C—H⋯O and C—H⋯N hydrogen bonds, and further extended into a three-dimensional network structure through inter­molecular aromatic π–π stacking inter­actions in which the centroid–centroid distance is 3.590 (1) Å.

## Related literature

For background to the applications of 2-oxazolidinones, see: Ippolito *et al.* (2008 ▶); Mullera *et al.* (1999 ▶).
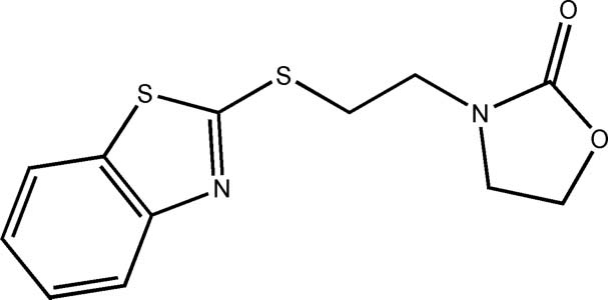


## Experimental

### 

#### Crystal data

C_12_H_12_N_2_O_2_S_2_
*M* *_r_* = 280.36
Triclinic, 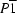

*a* = 6.5804 (4) Å
*b* = 7.8331 (5) Å
*c* = 12.5890 (7) Å
α = 99.864 (5)°
β = 97.715 (5)°
γ = 97.011 (5)°
*V* = 626.49 (7) Å^3^
*Z* = 2
Cu *K*α radiation
μ = 3.83 mm^−1^
*T* = 293 K
0.16 × 0.14 × 0.10 mm

#### Data collection

Oxford Diffraction Xcalibur Sapphire3 diffractometer
Absorption correction: multi-scan (*CrysAlis RED*; Oxford Diffraction, 2005 ▶) *T*_min_ = 0.572, *T*_max_ = 1.000
4029 measured reflections
2396 independent reflections
2081 reflections with *I* > 2σ(*I*)
*R*_int_ = 0.020

#### Refinement

*R*[*F* ^2^ > 2σ(*F* ^2^)] = 0.039
*wR*(*F* ^2^) = 0.111
*S* = 1.05
2396 reflections
163 parameters
H-atom parameters constrained
Δρ_max_ = 0.25 e Å^−3^
Δρ_min_ = −0.38 e Å^−3^

### 

Data collection: *CrysAlis CCD* (Oxford Diffraction, 2005 ▶); cell refinement: *CrysAlis CCD*; data reduction: *CrysAlis RED* (Oxford Diffraction, 2005 ▶); program(s) used to solve structure: *SHELXS97* (Sheldrick, 2008 ▶); program(s) used to refine structure: *SHELXL97* (Sheldrick, 2008 ▶); molecular graphics: *SHELXTL* (Sheldrick, 2008 ▶); software used to prepare material for publication: *OLEX2* (Dolomanov *et al.*, 2009 ▶).

## Supplementary Material

Crystal structure: contains datablocks I, global. DOI: 10.1107/S1600536810034264/bv2154sup1.cif

Structure factors: contains datablocks I. DOI: 10.1107/S1600536810034264/bv2154Isup2.hkl

Additional supplementary materials:  crystallographic information; 3D view; checkCIF report

## Figures and Tables

**Table 1:** Hydrogen-bond geometry (Å, °)

*D*—H⋯*A*	*D*—H	H⋯*A*	*D*⋯*A*	*D*—H⋯*A*
C11—H11*B*⋯O1^i^	0.97	2.58	3.466 (3)	152
C3—H3⋯O1^ii^	0.93	2.59	3.282 (2)	132
C5—H5⋯N1^i^	0.93	2.54	3.445 (2)	163

Symmetry codes: (i) 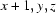; (ii) 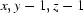.

**Fig. 1.**
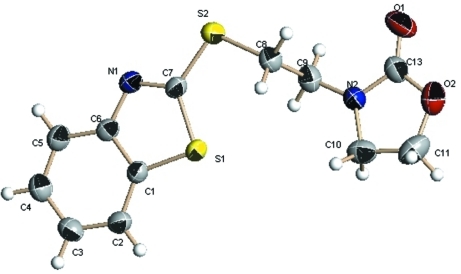
The structure of the title compound, showing the atom-labelling scheme. Displacement ellipsoids are drawn at the 30% probability level. H atoms have been omitted for clarity.

**Fig. 2.**
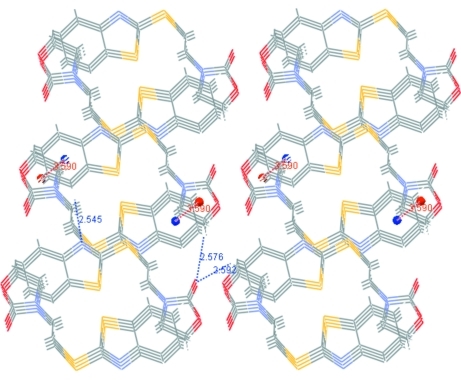
The three-dimensional structure by molecular packing, showing the hydrogen bonds as blue dashed lines, and π-π stacking interactions as red dashed lines.

## supplementary crystallographic information

### Comment

N-substituted 2-oxazolidinones have been widely used as antibiotics which are effective against gram-positive bacteria (Ippolito *et al.*, 2008; Mullera *et al.*, 1999). In this article we provide a new synthetic route of a 2-oxazolidinone derivative. Even though the reaction mechanism has not been established, the reproducibility and high yield of the reaction should prove useful for the synthesis of this type of compound.

Herein, we report the synthesis and structure of the title compound, namely 3-(2-(benzo[*d*]thiazol-2-ylthio)ethyl)-oxazolidin-2-one (Fig.1). As shown in Fig. 2, a two-dimensional supramolecular network was formed by hydrogen bonds (Table 1) and weak π-π stacking interactions between the phenyl rings and the thiazolyl rings of adjacent molecules with a centroid-centroid distances of 3.590 Å along *b* direction.

### Experimental

A mixture of 2-mercaptobenzothiazole (6.69 g, 0.04 mol), potassium carbonate (8.29 g, 0.06 mol) and ethanol (250 ml) was heated and stirred in a 500 ml flask. Bis(2-chloroethyl)amine hydrochloride (7.14 g, 0.04 mol, dissolved in 100 ml ethanol) was added dropwise into the flask when the mixture was heated to 353 K, and the mixture was further stired at 353 K for 8 h. After cooling, the precipitate was filtered, washed with ethanol and water, and recrystallized from ethanol to obtain a flaxen powder. Yield: 68%. ^1^H NMR (CDCl_3_, 400 MHz):3.60 (t, 2H), 3.74 (m, 4H), 4.3 (t, 2H), 7.45 (m, 2H), 7.77 (d, 1H), 7.79 (d, 1H). ^13^C NMR (CDCl_3_, 125 MHz): 31.17, 43.81, 45.58, 61.97, 121.40, 121.41, 124.55, 126.20, 135.26, 152.86, 158.40, 165.71.

### Refinement

The H atoms were placed at calculated positions in the riding model approximation (C—H 0.95–0.99 Å), with their temperature factors were set to 1.2 times those of the equivalent isotropic temperature factors of the parent atoms.

### Crystal data

**Table:**

C_12_H_12_N_2_O_2_S_2_	*Z* = 2
*M**_r_* = 280.36	*F*(000) = 292
Triclinic, *P*1	*D*_x_ = 1.486 Mg m^−^^3^
Hall symbol: -P 1	Cu *K*α radiation, λ = 1.54184 Å
*a* = 6.5804 (4) Å	Cell parameters from 2669 reflections
*b* = 7.8331 (5) Å	θ = 3.6–72.2°
*c* = 12.5890 (7) Å	µ = 3.83 mm^−^^1^
α = 99.864 (5)°	*T* = 293 K
β = 97.715 (5)°	Rhombus, colourless
γ = 97.011 (5)°	0.16 × 0.14 × 0.10 mm
*V* = 626.49 (7) Å^3^	

### Data collection

**Table:**

Oxford Diffraction Xcalibur Sapphire3 diffractometer	2396 independent reflections
Radiation source: Enhance (Cu) X-ray Source	2081 reflections with *I* > 2σ(*I*)
graphite	*R*_int_ = 0.020
Detector resolution: 16.0355 pixels mm^-1^	θ_max_ = 72.4°, θ_min_ = 3.6°
ω scans	*h* = −7→5
Absorption correction: multi-scan (*CrysAlis RED*; Oxford Diffraction, 2005)	*k* = −9→8
*T*_min_ = 0.572, *T*_max_ = 1.000	*l* = −15→15
4029 measured reflections	

### Refinement

**Table:**

Refinement on *F*^2^	Primary atom site location: structure-invariant direct methods
Least-squares matrix: full	Secondary atom site location: difference Fourier map
*R*[*F*^2^ > 2σ(*F*^2^)] = 0.039	Hydrogen site location: inferred from neighbouring sites
*wR*(*F*^2^) = 0.111	H-atom parameters constrained
*S* = 1.05	*w* = 1/[σ^2^(*F*_o_^2^) + (0.081*P*)^2^ + 0.008*P*] where *P* = (*F*_o_^2^ + 2*F*_c_^2^)/3
2396 reflections	(Δ/σ)_max_ < 0.001
163 parameters	Δρ_max_ = 0.25 e Å^−^^3^
0 restraints	Δρ_min_ = −0.38 e Å^−^^3^

### Special details

**Table:**

Geometry. All e.s.d.'s (except the e.s.d. in the dihedral angle between two l.s. planes) are estimated using the full covariance matrix. The cell e.s.d.'s are taken into account individually in the estimation of e.s.d.'s in distances, angles and torsion angles; correlations between e.s.d.'s in cell parameters are only used when they are defined by crystal symmetry. An approximate (isotropic) treatment of cell e.s.d.'s is used for estimating e.s.d.'s involving l.s. planes.
Refinement. Refinement of *F*^2^ against ALL reflections. The weighted *R*-factor *wR* and goodness of fit *S* are based on *F*^2^, conventional *R*-factors *R* are based on *F*, with *F* set to zero for negative *F*^2^. The threshold expression of *F*^2^ > σ(*F*^2^) is used only for calculating *R*-factors(gt) *etc*. and is not relevant to the choice of reflections for refinement. *R*-factors based on *F*^2^ are statistically about twice as large as those based on *F*, and *R*- factors based on ALL data will be even larger.

### Fractional atomic coordinates and isotropic or equivalent isotropic displacement parameters (Å^2^)

**Table:**

	*x*	*y*	*z*	*U*_iso_*/*U*_eq_	
S1	0.35142 (6)	0.68528 (6)	0.47728 (3)	0.04252 (17)	
S2	−0.03291 (7)	0.79117 (6)	0.57333 (4)	0.04677 (17)	
O1	0.2992 (3)	1.2011 (2)	0.90981 (13)	0.0693 (5)	
O2	0.6287 (3)	1.1616 (2)	0.95686 (12)	0.0661 (4)	
N1	−0.0218 (2)	0.6472 (2)	0.36963 (12)	0.0407 (3)	
N2	0.4734 (2)	1.0512 (2)	0.78894 (12)	0.0465 (4)	
C1	0.1020 (3)	0.5814 (2)	0.29599 (14)	0.0370 (4)	
C2	0.0334 (3)	0.5093 (3)	0.18583 (15)	0.0466 (4)	
H2	−0.1048	0.5029	0.1556	0.056*	
C3	0.1746 (3)	0.4474 (3)	0.12252 (15)	0.0500 (4)	
H3	0.1301	0.3982	0.0492	0.060*	
C4	0.3836 (3)	0.4578 (2)	0.16706 (16)	0.0474 (4)	
H4	0.4757	0.4152	0.1228	0.057*	
C5	0.4548 (3)	0.5301 (2)	0.27549 (15)	0.0424 (4)	
H5	0.5937	0.5382	0.3050	0.051*	
C6	0.3115 (3)	0.5908 (2)	0.33941 (14)	0.0358 (3)	
C7	0.0866 (3)	0.7046 (2)	0.46521 (14)	0.0370 (4)	
C8	0.1759 (3)	0.8424 (2)	0.68809 (14)	0.0441 (4)	
H8A	0.1174	0.8441	0.7549	0.053*	
H8B	0.2621	0.7503	0.6824	0.053*	
C9	0.3115 (3)	1.0177 (2)	0.69552 (15)	0.0474 (4)	
H9A	0.3729	1.0166	0.6296	0.057*	
H9B	0.2267	1.1109	0.7014	0.057*	
C10	0.6791 (3)	1.0058 (3)	0.78669 (19)	0.0533 (5)	
H10A	0.7520	1.0668	0.7389	0.064*	
H10B	0.6755	0.8806	0.7642	0.064*	
C11	0.7754 (4)	1.0685 (4)	0.9048 (2)	0.0683 (6)	
H11A	0.8023	0.9698	0.9390	0.082*	
H11B	0.9054	1.1453	0.9106	0.082*	
C12	0.4514 (3)	1.1428 (2)	0.88637 (15)	0.0480 (4)	

### Atomic displacement parameters (Å^2^)

**Table:**

	*U*^11^	*U*^22^	*U*^33^	*U*^12^	*U*^13^	*U*^23^
S1	0.0296 (2)	0.0517 (3)	0.0405 (3)	0.00659 (18)	−0.00164 (17)	−0.00180 (18)
S2	0.0372 (3)	0.0556 (3)	0.0451 (3)	0.0092 (2)	0.00809 (19)	−0.0001 (2)
O1	0.0739 (11)	0.0767 (11)	0.0617 (9)	0.0256 (9)	0.0276 (8)	0.0019 (8)
O2	0.0723 (10)	0.0682 (10)	0.0476 (8)	0.0023 (8)	−0.0068 (7)	0.0007 (7)
N1	0.0317 (7)	0.0481 (8)	0.0408 (8)	0.0079 (6)	0.0018 (6)	0.0062 (6)
N2	0.0426 (9)	0.0500 (8)	0.0431 (8)	0.0105 (7)	0.0052 (7)	−0.0030 (7)
C1	0.0337 (8)	0.0381 (8)	0.0395 (8)	0.0059 (6)	0.0031 (7)	0.0097 (7)
C2	0.0420 (10)	0.0542 (10)	0.0397 (9)	0.0055 (8)	−0.0022 (7)	0.0063 (8)
C3	0.0567 (11)	0.0524 (10)	0.0382 (9)	0.0068 (9)	0.0046 (8)	0.0043 (8)
C4	0.0509 (11)	0.0448 (9)	0.0485 (10)	0.0101 (8)	0.0163 (8)	0.0055 (8)
C5	0.0355 (9)	0.0411 (9)	0.0497 (10)	0.0065 (7)	0.0073 (7)	0.0055 (7)
C6	0.0325 (8)	0.0348 (7)	0.0380 (8)	0.0026 (6)	0.0021 (6)	0.0055 (6)
C7	0.0304 (8)	0.0379 (8)	0.0412 (9)	0.0049 (6)	0.0027 (7)	0.0055 (7)
C8	0.0489 (10)	0.0438 (9)	0.0393 (9)	0.0074 (8)	0.0074 (8)	0.0061 (7)
C9	0.0554 (11)	0.0409 (9)	0.0434 (9)	0.0079 (8)	0.0032 (8)	0.0044 (7)
C10	0.0478 (11)	0.0488 (10)	0.0680 (13)	0.0128 (8)	0.0168 (10)	0.0146 (9)
C11	0.0440 (12)	0.0807 (16)	0.0786 (16)	0.0031 (11)	−0.0035 (11)	0.0250 (13)
C12	0.0544 (11)	0.0448 (9)	0.0431 (10)	0.0062 (8)	0.0100 (8)	0.0028 (8)

### Geometric parameters (Å, °)

**Table:**

S1—C6	1.7376 (17)	C3—C4	1.401 (3)
S1—C7	1.7564 (17)	C3—H3	0.9300
S2—C7	1.7412 (17)	C4—C5	1.379 (3)
S2—C8	1.8083 (19)	C4—H4	0.9300
O1—C12	1.202 (2)	C5—C6	1.394 (2)
O2—C12	1.343 (2)	C5—H5	0.9300
O2—C11	1.439 (3)	C8—C9	1.525 (3)
N1—C7	1.290 (2)	C8—H8A	0.9700
N1—C1	1.389 (2)	C8—H8B	0.9700
N2—C12	1.346 (2)	C9—H9A	0.9700
N2—C9	1.441 (2)	C9—H9B	0.9700
N2—C10	1.444 (3)	C10—C11	1.509 (3)
C1—C2	1.396 (2)	C10—H10A	0.9700
C1—C6	1.401 (2)	C10—H10B	0.9700
C2—C3	1.382 (3)	C11—H11A	0.9700
C2—H2	0.9300	C11—H11B	0.9700
			
C6—S1—C7	88.66 (8)	C9—C8—S2	113.55 (13)
C7—S2—C8	103.28 (8)	C9—C8—H8A	108.9
C12—O2—C11	109.30 (16)	S2—C8—H8A	108.9
C7—N1—C1	110.61 (14)	C9—C8—H8B	108.9
C12—N2—C9	122.28 (17)	S2—C8—H8B	108.9
C12—N2—C10	112.88 (17)	H8A—C8—H8B	107.7
C9—N2—C10	124.61 (16)	N2—C9—C8	110.74 (16)
N1—C1—C2	125.28 (16)	N2—C9—H9A	109.5
N1—C1—C6	115.23 (15)	C8—C9—H9A	109.5
C2—C1—C6	119.48 (17)	N2—C9—H9B	109.5
C3—C2—C1	118.82 (18)	C8—C9—H9B	109.5
C3—C2—H2	120.6	H9A—C9—H9B	108.1
C1—C2—H2	120.6	N2—C10—C11	100.92 (18)
C2—C3—C4	121.05 (18)	N2—C10—H10A	111.6
C2—C3—H3	119.5	C11—C10—H10A	111.6
C4—C3—H3	119.5	N2—C10—H10B	111.6
C5—C4—C3	120.99 (18)	C11—C10—H10B	111.6
C5—C4—H4	119.5	H10A—C10—H10B	109.4
C3—C4—H4	119.5	O2—C11—C10	106.53 (17)
C4—C5—C6	117.81 (17)	O2—C11—H11A	110.4
C4—C5—H5	121.1	C10—C11—H11A	110.4
C6—C5—H5	121.1	O2—C11—H11B	110.4
C5—C6—C1	121.83 (16)	C10—C11—H11B	110.4
C5—C6—S1	128.83 (14)	H11A—C11—H11B	108.6
C1—C6—S1	109.34 (13)	O1—C12—O2	123.44 (19)
N1—C7—S2	119.85 (13)	O1—C12—N2	126.8 (2)
N1—C7—S1	116.15 (13)	O2—C12—N2	109.76 (17)
S2—C7—S1	123.99 (10)		
			
C7—N1—C1—C2	179.93 (17)	C8—S2—C7—S1	−1.39 (13)
C7—N1—C1—C6	0.0 (2)	C6—S1—C7—N1	0.16 (14)
N1—C1—C2—C3	−179.38 (17)	C6—S1—C7—S2	179.01 (12)
C6—C1—C2—C3	0.5 (3)	C7—S2—C8—C9	82.41 (15)
C1—C2—C3—C4	−0.6 (3)	C12—N2—C9—C8	−92.9 (2)
C2—C3—C4—C5	0.0 (3)	C10—N2—C9—C8	92.9 (2)
C3—C4—C5—C6	0.7 (3)	S2—C8—C9—N2	179.53 (12)
C4—C5—C6—C1	−0.7 (3)	C12—N2—C10—C11	6.1 (2)
C4—C5—C6—S1	179.22 (14)	C9—N2—C10—C11	−179.26 (19)
N1—C1—C6—C5	−179.97 (15)	C12—O2—C11—C10	7.0 (3)
C2—C1—C6—C5	0.1 (3)	N2—C10—C11—O2	−7.6 (2)
N1—C1—C6—S1	0.08 (19)	C11—O2—C12—O1	176.9 (2)
C2—C1—C6—S1	−179.82 (14)	C11—O2—C12—N2	−3.2 (2)
C7—S1—C6—C5	179.93 (17)	C9—N2—C12—O1	3.0 (3)
C7—S1—C6—C1	−0.13 (13)	C10—N2—C12—O1	177.8 (2)
C1—N1—C7—S2	−179.04 (12)	C9—N2—C12—O2	−176.91 (16)
C1—N1—C7—S1	−0.14 (19)	C10—N2—C12—O2	−2.1 (2)
C8—S2—C7—N1	177.42 (14)		

### Hydrogen-bond geometry (Å, °)

**Table:**

*D*—H···*A*	*D*—H	H···*A*	*D*···*A*	*D*—H···*A*
C11—H11B···O1^i^	0.97	2.58	3.466 (3)	152
C3—H3···O1^ii^	0.93	2.59	3.282 (2)	132
C5—H5···N1^i^	0.93	2.54	3.445 (2)	163

Symmetry codes: (i) *x*+1, *y*, *z*; (ii) *x*, *y*−1, *z*−1.
